# Injury Patterns in Vehicle Crashes: The Significance of Occupant Seating Position

**DOI:** 10.7759/cureus.53730

**Published:** 2024-02-06

**Authors:** Zachary Taylor, Austin Henken-Siefken, Andrew McCague

**Affiliations:** 1 Medicine, College of Osteopathic Medicine of the Pacific - Northwest, Western University of Health Sciences, Lebanon, USA; 2 General Surgery, Desert Regional Medical Center, Palm Springs, USA; 3 Surgery, Desert Regional Medical Center, Palm Springs, USA; 4 Trauma and Acute Care Surgery, Desert Regional Medical Center, Palm Springs, USA

**Keywords:** road traffic safety, motor vehicle passengers, motor vehicle drivers, motor vehicle accidents, injury severity

## Abstract

Objectives: Investigating patterns among the outcomes of patients involved in motor vehicle accidents (MVAs) can provide information necessary to guide targeted interventions to improve road traffic safety. Our purpose is to identify any differences between passenger and driver injury severity and overall clinical course after MVAs.

Methods: We performed a retrospective review and analysis of 3,693 patients involved in MVAs from 2016 to 2021. We divided the data into two groups, drivers and passengers, and compared the Injury Severity Score (ISS), Revised Trauma Score (RTS) on admission, days in the Intensive Care Unit (ICU), length of hospital stay (LOS), post Emergency Department (ED) disposition, discharge (DC) disposition, and signs of life on arrival (SOLA) to the ED. We compared mean ISS, New Injury Severity Score (NISS), RTS, length in ICU and LOS using a student’s T-test and SOLA, post-ED and DC disposition using Chi-square analysis.

Results: We did not find any statistically significant difference in ISS, RTS, days in ICU, LOS, or SOLA between the drivers and passengers. However, we did find a statistically significant difference in the post-ED (X^2^= 113.743, p=<0.0001) and DC disposition (X^2^=41.172, p=<0.0001) of drivers and passengers. After the ED and DC, more passengers were transferred to a higher level of care than expected, while the inverse was true for drivers. The number of drivers discharged to Skilled Nursing Facilities (SNFs) was also higher than expected, further contributing to the observed difference in DC disposition.

Conclusion: Our study found no statistically significant difference between driver and passenger injury severity, length of hospital stay, days in ICU, and SOLA after an MVA. The clinical courses of the two groups were found to be significantly different based on post-ED and DC disposition data. We identified limitations, such as a relatively small sample size and insufficient data on specific car seat locations for passengers, underscoring the need for a more nuanced exploration. Future research must broaden its scope to encompass diverse crash scenarios, vehicle design and safety technologies, seat belt dynamics, and age- and gender-specific vulnerabilities.

## Introduction

Motor vehicle accidents are a significant public health concern, contributing to numerous injuries and fatalities globally. The resulting injuries are estimated to claim 1.35 million lives each year and are the worldwide leading cause of death in children and young adults aged 5 to 29 years old [1]. Motor vehicle injury patterns and how their likelihood and severity relate to car occupant position, are a subject of evolving research. Current literature has shown an increased risk of injury associated with both front and rear passengers when compared to drivers, indicating a potential discrepancy in injury severity based on seating position [2,3]. Understanding the patterns of injuries suffered by occupants in car accidents is critical for developing effective safety measures and interventions. This research paper aims to investigate the difference, if any, in the injury severity and overall clinical course between drivers and passengers involved in motor vehicle accidents (MVAs). 

## Materials and methods

We obtained and retrospectively analyzed the data of patients who presented to our level I trauma center after an MVA from 2016 to 2021. The data was pulled from our ImageTrend trauma registry, which is updated by our certified trauma registrars. This de-identified data set included the following: demographic data, Injury Severity Score (ISS), Revised Trauma Score (RTS) on admission, signs of life on arrival (SOLA), days in ICU and length of hospital stay, post-emergency department (ED) disposition, discharge (DC) disposition. We chose to include patients with documentation of each of these parameters because we believe they provide an adequate indication of patients’ injury severity and overall clinical course. The ISS was calculated by first examining injuries in six body regions of each patient (head or neck, face, chest, abdomen or pelvis, extremities and external), where each region was given an Abbreviated Injury Scale (AIS) score based on the severity of the injury. The AIS scores of the three most severely injured regions were squared and added together to find the ISS. The RTS was calculated by assigning a numerical value to each patient's Glasgow Coma Scale (GCS) score, systolic blood pressure (SBP), and respiratory rate (RR). We then calculated the weighted sum of these scores using the formula RTS = (0.9368*GCS Value)+(0.7326*SBP Value)+(0.2908*RR Value) to find the RTS [4]. Also, we included patients recorded in the dataset as the driver or passenger of the crashed vehicle, however, the dataset did not specify which seat, rear or front, passengers were in, nor information regarding seatbelt use. We divided the data into two groups, drivers and passengers, then examined the parameters and compared them across the two groups. We excluded patients whose role as driver or passenger was not specifically documented and patients who left against medical advice (AMA).

Statistical analysis was performed using SAS Studio statistical software, version 3.8 (SAS Institute Inc., Cary, NC). We performed a student’s T-test to compare ISS, RTS, days in ICU, and LOS and Chi-square analysis to compare mortality, post-ED and DC disposition. The presence of SOLA in the ED, post-ED and DC disposition were analyzed with Chi-square tests. Signs of life were defined as pupillary response, spontaneous ventilation, presence of carotid pulse, measurable blood pressure, extremity movement, or cardiac electrical activity. Statistically significant chi-square tests were followed by an analysis of residuals, or the difference between observed and expected values, to determine which variables contributed most to the result. We calculated the adjusted residuals to adjust for the sample size by taking the residuals and dividing them by the standard error. A positive adjusted residual value indicated a higher frequency was observed than expected, while a negative value indicated a lower frequency was observed than expected. A p-value of less than 0.05 and an absolute adjusted residual value of 2 or greater were considered statistically significant.

## Results

The data set we analyzed included 3,693 patients involved in an MVA and 3,608 patients met the necessary analysis criteria. Of these patients, 2,605 (72%) were drivers and 1,002 (28%) were passengers in either the front seat or a rear seat. There were more males than females in both groups. We also found a much higher proportion of pediatrics in the passenger group, which was expected. See Table 1 for demographic data. 

**Table 1:** Demographics of drivers and passengers

	Driver	Passenger
Male	1,534	391
Female	1,062	607
Sex unknown	9	4
Ages (0-17 years)	41	182
18-29 years	824	281
30-49 years	734	224
50-64 years	464	163
65+ years	542	150

The average ISS, length of hospital stay, Revised Trauma Score (RTS) on admission, and length of ICU stay between drivers and passengers were compared and analyzed with a student’s T-test. See Table 2 for the comparison of means between the groups. We found no statistically significant difference for any of these parameters. 

**Table 2:** Mean ISS, LOS, RTS on admission and ICU days for drivers and passengers analyzed by student's T-test ISS, Injury Severity Score, Range: 0-75; LOS, Length of hospital stay; RTS, Revised Trauma Score, Range: 0-7.84; ICU Days, days spent in the Intensive Care Unit (ICU).

Parameter	Driver	Passenger	P value
ISS	7.913	8.069	.6152
LOS (days)	6.873	6.692	.647
RTS on Admission	7.754	7.739	.401
ICU Days	1.556	1.355	.215

We performed a Chi-square analysis comparing the presence of SOLA to the ED after an MVA to the patients’ seat positions. There was no statistically significant difference between SOLA for drivers and passengers, X^2^ (degrees of freedom (DF) = 1, N = 3,596) = 0.01, p = 0.925. See Table 3.

**Table 3:** Comparison of signs of life on arrival to the ED between drivers and passengers Data were presented as N(%). ED, Emergency Department.

Signs of Life on Arrival	Driver	Passenger	Total
Yes	2577 (71.66)	991 (27.56)	3568 (99.22)
No	20 (0.56)	8 (0.22)	28 (0.78)

We also used a Chi-square analysis to examine the post-ED disposition of 3,494 drivers and passengers, excluding patients who left against medical advice (AMA). We found that the differences in post-ED disposition between drivers and passengers were statistically significant, X^2^ (DF = 6, N = 3,494) = 113.743, p = <0.0001. See Table 4. We then examined the residuals of the Chi-square test, see Table 5.

**Table 4:** Chi-square test of post-emergency department disposition between drivers and passengers Data were presented as N(%).

Post Emergency Department Disposition	Driver	Passenger	Total
Home	928 (26.56)	338 (9.67)	1266 (36.23)
Admitted to intensive care unit	535 (15.31)	181 (5.18)	716 (20.49)
Admitted to another inpatient facility	873 (24.99)	183 (5.24)	684 (19.58)
Transferred to a higher level of care	9 (0.26)	55 (1.57)	64 (1.84)
Morgue (expired in the emergency department)	26 (0.74)	9 (0.26)	35 (1.00)
Operating room	141 (4.04)	69 (1.97)	210 (6.01)
Skilled nursing facility	2 (0.06)	0	2 (0.06)

**Table 5:** Comparison of driver and passenger post-emergency department disposition residuals Adj*. *Res, Adjusted Residual; 'Observed' refers to the number of patients in the column title category;​​​​​​​ 'Expected' refers to the number of patients expected in the absence of association between the variables;​​​​​​​ 'Total' refers to the sum of all patients in each category.

		Post Emergency Department Disposition							
Patient		Home	Admitted to intensive care unit	Admitted to another inpatient facility	Transferred to a higher level of care	Morgue (expired in the emergency department)	Operating room	Skilled nursing facility	Total
Driver	Observed	928	535	873	9	26	141	2	2514
	Expected	910.91	515.18	864.14	46.049	25.183	151.1	1.439	
	Residual	17.089	19.824	8.858	-37.05	0.817	-10.1	0.561	
	Adj. Res	1.339	1.850	0.7023	-10.405	0.309	-1.60	0.883	
Passenger	Observed	338	181	328	55	9	69	0	980
	Expected	355.09	200.82	336.86	17.951	9.8168	58.901	0.561	
	Residual	-17.089	-19.824	-8.858	37.05	-0.817	10.1	-0.561	
	Adj. Res	-1.339	-1.850	-0.702	10.405	-0.309	1.60	-0.883	
	Total	64	1201	1266	716	35	210	2	3494

The adjusted residual value of drivers transferred to a higher level of care (-10.405) implies that there were significantly fewer drivers transferred to a higher level of care after the ED than expected. On the other hand, the passenger-adjusted residual value (+10.405) indicates that there were significantly more passengers transferred to a higher level of care after the ED than expected. 

Lastly, we analyzed the DC locations of 2,563 drivers and passengers using Chi-square analysis and found the differences between driver and passenger DC disposition were statistically significant, X^2^ (DF = 5, N = 2,563) = 41.172, p = <0.0001, see Table 6. We also examined the residuals in Table 7.

**Table 6:** Chi-square test of discharge disposition between drivers and passengers. Data were presented as N(%). SNF, Skilled Nursing Facilities.

Discharge Disposition	Driver	Passenger	Total
Home	1384 (54.00)	538 (20.99)	1922 (74.99)
Transferred to a higher level of care	38 (1.48)	48 (1.87)	86 (3.36)
Long term care	37 (1.44)	10 (0.39)	47 (1.83)
Inpatient rehabilitation	125 (4.88)	45 (1.76)	170 (6.63)
SNF	194 (7.57)	51 (1.99)	245 (9.56)
Morgue (Expired after emergency department disposition)	63 (2.46)	30 (1.17)	93 (3.63)

**Table 7:** Comparison of driver and passenger discharge disposition Chi-square residuals Adj. Res, Adjusted Residual; rehab, Rehabilitation; 'Observed' refers to the number of patients in the column title category;​​​​​​​ 'Expected' refers to the number of patients expected in the absence of association between the variables; ​​​​​​​'Total' refers to the sum of all patients in each category.

		Discharge Disposition						
Patient		Home	Transferred to a higher level of care	Long term care	Inpatient rehab	Morgue (Expired after emergency department disposition)	Skilled nursing facility	Total
Driver	Observed	1384	38	37	125	63	194	1841
	Expected	1380.6	61.774	33.76	122.11	66.802	175.98	
	Residual	3.40	-23.774	3.24	2.890	-3.802	18.017	
	Adj. Res	0.348	-5.797	1.06	0.510	-0.893	2.691	
Passenger	Observed	538	48	10	45	30	51	722
	Expected	541.43	24.226	13.24	47.889	26.198	69.017	
	Residual	-3.43	23.774	-3.24	-2.889	3.802	-18.17	
	Adj. Res	-0.348	5.797	-1.06	-0.510	0.893	-2.691	
	Total	86	1922	47	93	170	245	2563

Residual analysis of DC disposition revealed that the difference in drivers and passengers discharged to a higher level of care and SNFs both significantly contributed to the difference in DC disposition. The adjusted residual for drivers transferred to a higher level of care on DC (-5.797) indicates that significantly fewer drivers were transferred than expected, as opposed to the passenger adjusted residual (+5.797), which indicates that significantly more passengers were transferred than expected. In terms of discharge to SNFs, significantly more drivers were discharged to SNFs than expected (Adj. Res = 2.691), while the inverse proved to be true for passengers (Adj. Res = -2.691).

## Discussion

Motor vehicle accidents (MVAs) and associated factors are subjects of a myriad of ongoing research, given their significant contribution to injury, mortality, and strain on emergency healthcare systems. In 2021, the United States saw 2.5 million people injured in MVAs and 42,939 deaths associated with MVAs [5]. This prevalence has led to a substantial burden on emergency services, not only in the U.S. but globally [6]. Therefore, it is imperative to develop sustainable analyses of injury severity and overall clinical courses of both drivers and passengers after MVAs to find patterns and ensure more efficient, effective care.

Patients who experience MVAs present with a complex landscape of injuries, both drivers and passengers. Understanding disparities in injury severity and mortality between passengers and drivers can help guide more targeted interventions. 

The seating position within a vehicle has been identified as a critical factor influencing injury severity and mortality in car accidents [2,3,7]. Current trends in literature seem to point toward passengers, both front and back seats, having less injury severity and overall mortality than drivers. A matched-pair analysis, that considered variables such as age, sex, and restraint use has found that passengers, particularly those in the front right seat, exhibit a slightly higher risk of severe injuries and fatality compared to drivers in certain scenarios [7]. A study of 28,653 trauma patients found that front-seat passenger mortality was 0.47% higher than in drivers and that both front and back-seat passengers were more likely to suffer from traumatic brain injury (TBI) serious abdominal injuries [2]. Another study on 4,189 drivers and 954 front-seat passengers also demonstrated a higher injury severity and mortality rate in front-seat passengers [3]. Further comparing only front and back seat passengers, a study on 25,230 patients who experienced an MVA showed that the mortality rate of rear-seat passengers was 39% less than that of front-seat passengers [8]. One plausible explanation for the observed differences in injury severity could be due to the specific vulnerabilities faced by front-seat passengers. The absence of control mechanisms, such as the steering wheel, might render passengers more susceptible to certain injury mechanisms. The use of restraints also plays a pivotal role in influencing severity outcomes. Thus, if passengers, especially those in the front right seat, are less likely to use seat belts consistently, it could contribute significantly to the increased risk of severe injuries [7].

Other research has demonstrated the protective factors associated with certain passenger seats as well. A matched cohort study using the Fatality Analysis Reporting System (FARS) looked at how seating position, age, restraint use, and airbag presence impacted the risk of death within 30 days of a crash. This study revealed that, on average, rear-seat passengers have a 21% lower risk of death compared to front-seat passengers. Variations in the association between rear-seat passenger status and a lower risk of death were further explored based on age, restraint use, and airbag presence, emphasizing the protective effect of rear seating, particularly for children [9]. Research has further investigated which specific rear seat is safer, and the rear middle seat has been shown to be associated with a higher chance of survival and lower injury severity than all other seats. Occupants in the rear middle seat have 29.1% increased odds of survival over the first-row seating positions. Researchers further compared the rear middle seat to other rear seats and found 25% increased odds of survival in the rear middle seat. These results held true even after correcting for potential confounders [10]. The identification of a safer seating position can inform not only individual choices but also have broader implications for vehicle design and safety regulations. 

Ultimately, multiple complex factors contribute to differences in injury severity and mortality between drivers and passengers. As we have shown in our study, passengers may require a higher level of care more often than drivers, both after the ED and after hospital DC. Other studies point to possible explanations for such a discrepancy based on factors such as specific seating position, restraint use, and the specific mechanics of a car accident, as these all lead to variations in outcomes. A comprehensive approach to addressing these differences requires targeted interventions that focus on the specific vulnerabilities faced by drivers and passengers in MVAs. 

Our study solely differentiated driver status from passenger status without specific information regarding passenger seating position, i.e., front or rear seat, as we did not have access to this information in our dataset. We did not find any statistically significant differences between drivers and passengers in terms of injury severity or signs of life on arrival, but we did find some statistically significant evidence that the two groups have different post-ED and DC dispositions. Although correlational strength is low, this information has significant implications for the overall clinical courses of drivers and passengers. The difference in number of drivers and passengers transferred to a higher level of care after DC and the ED contributed the most to the difference in disposition. More passengers were transferred to a higher level of care than expected, while the opposite was found for drivers. We speculate this may be associated with specific injury patterns experienced by passengers, seatbelt compliance, and the fact that the passenger group has a larger pediatric population, thus more vulnerable patients. We also found that significantly more drivers and less passengers were sent to SNFs than expected after DC. This may imply that drivers can rehabilitate their MVA injuries in settings of less intensive acute care (i.e., SNFs) more often than passengers who, as previously shown, more often require a higher level of care. Our findings can guide further exploration into the development of targeted treatment and emergency response protocols for drivers and passengers. There are many other underlying factors that may also contribute to the observed differences in disposition as well, paving new avenues for future research.

The major limitations of this study are the relatively small sample size and the lack of data regarding the specific car seat location of passengers at the time of the incident. Having a larger sample size and specific seat information would allow us to better generalize our findings and expand our research from the difference between drivers and passengers to differences between passengers in different seats as well. Considering the potential differences in injury patterns based on specific seating positions, future studies should incorporate this level of detail. We also did not have access to information regarding the types of vehicles, speed of the vehicles upon impact, or seat belt use; all of which are confounding variables that reduce the generalizability of our study.

Future research should investigate how different accident types, such as side and rear impacts or rollovers, contribute to differences in injury patterns and mortality between drivers and passengers. Especially as both accident type and mode have been found to have substantial effects on the relative risk of death due to ejection from vehicles [11]. Furthermore, automatic emergency braking systems, collision avoidance systems, and other advanced vehicle safety technologies are also an evolving area of interest. Automatic Emergency Braking Systems have already been predicted to greatly improve road traffic safety in China [12]. The integration of these technological aspects into research may elucidate their role in mitigating injury severity and mortality discrepancies between drivers and passengers. The dynamics of consistent seat belt use among passengers and drivers, specifically focusing on compliance rates, is also an intriguing area of future research. Identifying patterns in seatbelt noncompliance can direct road safety initiatives to the audiences that may benefit from them the most. Research has shown that the prevalence of seat belt use is highest in drivers, followed by front seat passengers, and lastly rear seat passengers. Furthermore, women drivers, on average, used seatbelts more often than men drivers [13]. Understanding factors that influence seat belt compliance, such as passenger demographics and behavioral aspects, could also inform targeted interventions to enhance overall safety. Investigation into the association between vehicle design and injury outcomes is also warranted as differences in vehicle weight and design have been shown to influence occupant safety [14]. Further assessment of how structural reinforcements in vehicles influence injury severity and mortality would contribute to optimizing vehicle safety. The intersectionality of demographic factors also calls for focused research. A 13-year cohort study showed that while men are more likely to crash a vehicle, women are at a higher risk of MVA-related injury requiring hospitalization [15]. A significantly higher mortality rate among elderly people as compared to non-elderly people after an MVA suggests that age also plays a role in MVA-associated injury [16]. Building upon the investigation of age and gender-specific vulnerabilities and how physiological factors are associated with injury severity and mortality could guide age and gender-appropriate safety recommendations and interventions.

## Conclusions

The prevalence of injuries and fatalities associated with MVAs underscores the need for sustainable analyses of injury severity and clinical outcomes for both drivers and passengers. Understanding the disparities in injury severity and clinical course between these two groups is crucial for guiding targeted interventions. Our study did not find any statistically significant differences in driver and passenger injury severity, length of hospital stay, days in ICU, and SOLA after an MVA; however, we found a statistically significant difference in their post-ED and DC disposition. A relatively small sample size and lack of data regarding specific passenger seat positions at the time of incident limited our ability to extrapolate our data to a larger population and contribute to current research involving seat position-specific research. Future research should include information on different crash scenarios, vehicle design and safety technologies, seat belt compliance, and age- and gender-specific vulnerabilities to advance our understanding and guide interventions to enhance road safety outcomes on a global scale.
